# The *MLH1 *2101C>A (Q701K) variant increases the risk of gastric cancer in Chinese males

**DOI:** 10.1186/1471-230X-11-133

**Published:** 2011-12-03

**Authors:** Wenxian Zhi, Binshuang Xue, Lifeng Wang, Nong Xiao, Qiong He, Yaping Wang, Yimei Fan

**Affiliations:** 1 Department of Medical Genetics, Medical School, Nanjing University, Nanjing, China; 2 Jiangsu Key Laboratory of Molecular Medicine, Nanjing, China; 3 Department of Oncology, Drum Tower Hospital Affiliated to Medical School, Nanjing University, Nanjing, China; 4 Lujiang Hospital, Anhui, China

## Abstract

**Background:**

Gastric cancer is one of the most common cancers affecting East Asians, and *MLH1 *could play a critical role during tumorigenesis in this condition.

**Methods:**

Samples from 236 Chinese patients suffering from gastric cancer were screened for *MLH1 *germline mutations. Carrier frequencies of the mutations were compared between gastric cancer patients and 240 cancer-free controls. Bioinformatic analysis was used to predict the effect of these mutations on protein function and mRNA splicing.

**Results:**

Six *MLH1 *sequence alterations were identified in gastric cancer patients including two promoter region substitutions, -93G>A and -28A>G, and four missense mutations 649C>T (R217C), 655A>G (I219V), 1151T>A (V384D) and 2101C>A (Q701K). Compared with the *MLH1 *2101CC genotype, the 2101CA genotype was associated with a risk of gastric cancer (OR = 8.42, 95% CI = 1.04-68.06) in males. Furthermore, the *MLH1 *2101C>A mutant was predicted by *in silico *analysis to affect exon splicing ability. Immunohistochemistry of one index patient carrying the *MLH1 *2101C>A mutation demonstrated a loss of MLH1 protein and normal expression of MSH2 and E-cadherin. No significant differences were demonstrated between cases and controls for the other five *MLH1 *variants but the data indicated an ethnic difference in the frequency of these variations between Eastern Asians and Western populations.

**Conclusions:**

An ethnic-specific *MLH1 *mutation spectrum occurred in Chinese gastric cancer patients. The *MLH1 *2101C>A mutation could be a marker for susceptibility to gastric cancer, particularly in males.

## Background

Gastric cancer is one of the most common malignancies worldwide and is the leading cancer in East Asian countries [1]. There are two histopathological types of gastric cancer, differentiated and undifferentiated [2], or intestinal and diffuse types [3]. Environmental and genetic factors may play important roles in this condition and in order to understand its etiology, several genes have been analyzed but few variation genotypes have been identified. Intestinal gastric cancers have been identified as common extracolonic tumors in the hereditary nonpolyposis colorectal cancer (HNPCC) syndrome [1], which are often caused by germline mutations of mismatch repair genes, predominantly *MLH1 *(Gene ID 4292)[4]. Several groups have investigated the association between *MLH1 *mutations and the risk of developing several cancer types including colorectal and lung cancer. However, mutations of *MLH1 *and their association with gastric cancer are rarely studied. It is possible that some *MLH1 *mutations could have an effect on mismatch repair functions, thereby modulating the susceptibility to the condition. To clarify the significance of *MLH1 *mutations in the development of gastric cancer, a study was carried out in 236 Chinese gastric cancer patients to achieve a full spectrum of germline *MLH1 *mutations. In addition, a case-control study was carried out to investigate the association between the mutations and gastric cancer. Furthermore, bioinformatic analysis was used to predict the effect of these substitutions on protein function and mRNA splicing.

## Methods

### Clinical samples

Gastric cancer patients with onset from January 1 to December 31 of 2008, from the East District of China, whose tumors had been confirmed using histology, were investigated (178 men and 58 women, mean age 62.3 ± 9.4 years, range 30-84). A total of 240 cancer-free controls were recruited (mean age 61.8 ± 10.1 years, range 26-82) (Table 1). Details regarding gastric cancer family history, onset age and histological classification are summarized in Table 1. Informed consent was obtained from all subjects who underwent genetic testing, according to the Ethics Committee of the Medical School of Nanjing University.

**Table 1:** Frequency distributions of variables in gastric cancer cases and controls.

Variables	Cases	Controls	*P*^a^
Number	236	240	
Age (years)			0.997
≤ 49	19 (8.1%)	19 (7.9%)	
50-59	65 (27.5%)	65 (27.1%)	
60-69	94 (39.8%)	98 (40.8%)	
≥ 70	58 (24.6%)	58 (24.2%)	
Gender			0.915
Male	178 (75.4%)	180 (75.0%)	
Female	58 (24.6%)	60 (25.0%)	
Family history			
Familial recurrence for gastric cancer (F)^b^	6 (2.5%)		
Low familial recurrence for gastric cancer (LF)^c^	39 (16.5%)		
Young age (< 50) of sporadic disease (Y)	16 (6.8%)		
Old age (≥ 50) of sporadic disease (S)	175 (74.2%)		
Tumor type (Available for 162 cases)			
Poorly differentiated	64 (39.5%)		
Moderately differentiated	69 (42.6%)		
Well differentiated	29 (17.9%)		

^a ^Two-sided χ2 test;

^b ^Individuals with gastric cancer and two or more first-degree relatives with gastric cancer or related cancers;

^c ^Individuals with gastric cancer and one first-degree relative with gastric cancer or related cancers.

### Immunohistochemistry analysis

Immunohistochemistry (IHC) of MLH1, MSH2 and E-cadherin was performed using formalin-fixed, paraffin-embedded tissue sections. Tissues were stained with MLH1 antibody [Mouse monoclonal, G168-728 diluted 1:100; Zymed Laboratories, San Francisco, CA, USA], MSH2 antibody [Mouse monoclonal, G219-1129 diluted 1:100; Zymed] and E-cadherin antibody [Mouse monoclonal, 4A2C7 diluted 1:100; Zymed], and detected by the EnVision System (Dako, Carpinteria, CA, USA).

### Mutation screening

Genomic DNA was extracted from peripheral blood leukocytes using the QIAamp DNA Blood Mini Kit (Qiagen, Hilden, Germany) according to the manufacturer's instructions. Mutation screening of *MLH1 *exons 1-19 and neighboring intronic sequences was performed using polymerase chain reaction (PCR) and high-resolution melting (HRM) analysis, using a LightScanner system (Idaho technology, Salt Lake City, UT, USA). The samples that presented abnormal profiles were sequenced on an ABI 3130-Avant automated sequencer (Applied Biosystems, Foster City, CA, USA). The *MLH1 *promoter region was genotyped using PCR and directly sequenced on the ABI 3130-Avant automated sequencer. PCR conditions were as follows: 95°C for 30 seconds, 52-60°C for 30 seconds, and 72°C for 40 seconds for 35 cycles, followed by 72°C for 7 min. The PCR primers for amplification of the *MLH1 *gene were as described in the literature [5] with a minor modification (Additional file 1, Table S1).

### Bioinformatic analysis of MLH1 variants

The impact of amino acid allelic variants on protein structure/function can be predicted via analysis of multiple sequence alignments and protein 3D-structures. The Sorting Intolerant from Tolerant (SIFT) algorithm was applied. SIFT is a program that predicts the effect of amino acid substitutions on protein function, on the basis of sequence conservation during evolution and the nature of the amino acids substituted in a gene of interest. SIFT scores were calculated online http://sift.jcvi.org/. If the value is less than 0.05, the amino acid substitution is predicted as intolerant, while those with a value greater than or equal to 0.05 are classified as tolerated.

Changes in exonic splicing enhancers (ESEs) due to single base substitutions were calculated using the ESEfinder program http://rulai.cshl.edu/tools/ESE. ESEfinder provides a score matrix based on the frequencies of the individual nucleotides at each position of the motif sequences specifically recognized by four SR proteins: SF2/ASF, SC35, SRp40 and SRp55 [6].

### Statistical analysis

χ2 tests or Fisher's exact test were used to compare the distribution of variables between cases and controls. The Hardy-Weinberg equilibrium (p2+2pq+q2 = 1), where p is the frequency of the variant allele and q = 1-p, was tested by a goodness-of-fit χ2 test to compare the observed genotype frequencies with the expected genotype frequencies in cancer-free controls. Unconditional logistical regression analysis was used to calculate odds ratios (ORs) and their 95% confidence intervals (CIs) adjusted by age and gender. Mantel-Haenszel χ2 analysis was used to evaluate the effect of the *MLH1 *2101C>A genotype stratified by age or gender. All statistical tests were two-sided, with a *P *value of 0.05 considered significant, using SPSS software (version 16).

## Results

### Characteristics of the study population

The study comprised 236 gastric cancer cases and 240 cancer-free controls. There were no significant differences in the distributions of age and gender between the cases and controls (*P *= 0.997 and 0.915, respectively) (Table 1). The majority of studied cases were sporadic; approximately 20% had a family history of cancer. Tumor type was assessed in 162 cases and more than 80% of the cases were poorly differentiated or moderately differentiated adenocarcinoma (Table 1).

### MLH1 variations identified in gastric cancer patients

Six variations were identified in gastric cancer patients. Two of the variants were located in the *MLH1 *promoter region: -93 G>A (rs1800734) and -28A>G; four were missense mutations in the coding region: 649C>T (R217C) and 655 A>G (I219V, rs1799977) in exon 8, 1151T>A (V384D) in exon 12 and 2101C>A (Q701K) in exon 18. No frameshift or nonsense mutations were identified (Table 2).

**Table 2:** *MLH1 *mutations detected in gastric cancer cases compared with controls.

Genotypes	Cases	Controls	*P*^a^	OR (95% CI) ^b^	*P*^b^
-93 G>A			0.736		
GG	36 (15.3%)	42(17.5%)		1.00	
GA	111 (47.0%)	114(47.5%)			
AA	89 (37.7%)	84(35.0%)			
GA+AA	200 (84.7%)	198(82.5%)		1.18 (0.72-1.92)	0.510
A allele	61.2%	58.8%	0.435		
-28A>G			1.000^c^		
AA	232 (98.3%)	236 (98.3%)		1.00	
AG	4 (1.7%)	4 (1.7%)		1.02 (0.25-4.14)	0.978
G allele	0.85%	0.83%	1.000 ^c^		
649C>T (R217C)			0.120 ^c^		
CC	231 (97.9%)	239 (99.6%)		1.00	
CT	5(2.1%)	1 (0.4%)		5.25 (0.61-45.45)	0.132
T allele	1.1%	0.2%	0.121 ^c^		
655A>G (I219V)			0.257		
AA	229 (97.0%)	228 (95.0%)		1.00	
AG	7 (3.0%)	12 (5.0%)		0.58 (0.22-1.50)	0.262
G allele	1.5%	2.5%	0.262		
1151T>A (V384D)			1.000 ^c^		
TT	232 (98.3%)	236 (98.3%)			
TA	4 (1.7%)	4 (1.7%)		1.02 (0.25-4.15)	0.976
A allele	0.85%	0.83%	1.000 ^c^		
2101C>A (Q701K)			0.120		
CC	228 (96.6%)	237 (98.8%)		1.00	
CA	8 (3.4%)	3 (1.3%)		2.77 (0.73 -10.57)	0.136
A allele	1.7%	0.6%	0.122		

^a ^Two-sided χ 2 test for either genotype distribution or allele frequency.

^b ^Unconditional logistic regression adjusted by age and gender

^c ^Fisher's exact test

The variants are all in Hardy-Weinberg equilibrium in cancer-free controls. The goodness-of-fit χ2 test values for -93G>A, -28A>G, 649C>T, 655 A>G, 1151T>A and 2101C>A are 0.099, 0.017, 0.001, 0.158, 0.017 and 0.009, respectively. The *P *values are all larger than 0.05.

### MLH1 genotypes and risk of gastric cancer

The genotype and allele distributions of the six *MLH1 *variations between the cases and controls are summarized in Table 2. Unconditional logistical regression analysis demonstrated that *MLH1 *2101CA was not associated with a significantly elevated risk of gastric cancer (*P *= 0.136, Table 2), but further stratified analysis by gender revealed that the risk associated with this variant genotype was significant in males (OR = 8.42, 95% CI = 1.04-68.06; *P *= 0.041). In a sub-group of subjects aged between 50 and 59 years, there were more *MLH1 *2101CA genotypes in the cases (7.7%) than in control subjects (0.0%), but the difference was not significant (*P *= 0.069) (Table 3). A higher frequency of *MLH1 *649 T allele was detected in gastric cancer patients than in controls (1.1% and 0.2%, respectively; *P *= 0.121). The OR for heterozygote CT was 5.25 (95% CI = 0.61-45.45), but the difference was not significant (*P *= 0.132) (Table 2). No difference was demonstrated between the cases and controls for the two variants in the promoter region, -93 G>A and -28A>G, separated or combined (Table 2 and 4). No significant differences existed between the cases and controls for the remaining variations detected (Table 2).

**Table 3:** Stratification analysis of gastric cancer risk associated with the *MLH1 *2101C>A genotype frequencies.

	Cases		Controls		OR	(95%CI)	
Variables	CC	CA	CC	CA	CC	CA	*P*^a^
Age (years)							
≤ 49	19(100.0%)	0(0.0%)	19(100.0%)	0(0.0%)	1.00	-	-
50-59	60(92.3%)	5(7.7%)	65(100.0%)	0(0.0%)	1.00	-	0.069
60-69	93(98.9%)	1(1.1%)	96(98.0%)	2(2.0%)	1.00	0.52(0.05-5.79)	0.971
≥ 70	56(96.6%)	2(3.4%)	57(98.3%)	1(1.7%)	1.00	2.04(0.18-23.09)	1.000
Gender							
Male	170(95.5%)	8(4.5%)	179(99.4%)	1(0.6%)	1.00	8.42(1.04-68.06)	0.041
Female	58(100.0%)	0(0.0%)	58(96.7%)	2(3.3%)	1.00	-	0.493

^a ^Mantel-Haenszel χ2 test

**Table 4:** Pairwise joint association for *MLH1 *SNPs -93 G>A and -28A>G and gastric cancer risk.

Genotype 1	Genotype 2	Cases	Controls	OR	95% CI	*P*
-28A>G	-93 G>A					
AA	GG	36	42	1.00		
AG	GG	0	0	-	-	-
AA	GA or AA	196	194	1.18	0.72-1.92	0.59
AG	GA or AA	4	4	1.17	0.27-5.00	0.87

### Prediction of MLH1 protein activity and structure

The SIFT score for the MLH1 variants demonstrated that the R217C and V384D are sorted as intolerant, suggesting that these amino acid substitutions are predicted to damage protein function. The remaining two variants of *MLH1*, I219V and Q701K, are sorted as tolerant (Table 5).

**Table 5:** *MLH1 *missense mutations analyzed by SIFT.

Sequence variant	Structural alteration	SIFT scores	Prediction
649C>T	R217C	0.00	Intolerant
655 A>G	I219V	0.57	Tolerated
1151T>A	V384D	0.00	Intolerant
2101C>A	Q701K	0.76	Tolerated

### Prediction of exon splicing

The 2101C>A variant is located at an exon-intron boundary. ESEfinder predicted that the 2101C>A alteration reduced the positive score obtained for one SF2/ASF (2.68) to below the threshold, while it increased another SF2/ASF motif (2.10/3.09). It produced a new positive score for SRp40 (3.68) but decreased another SR protein, SC35 motif (3.97/3.59).

### Protein expression analysis

**Figure 1:**
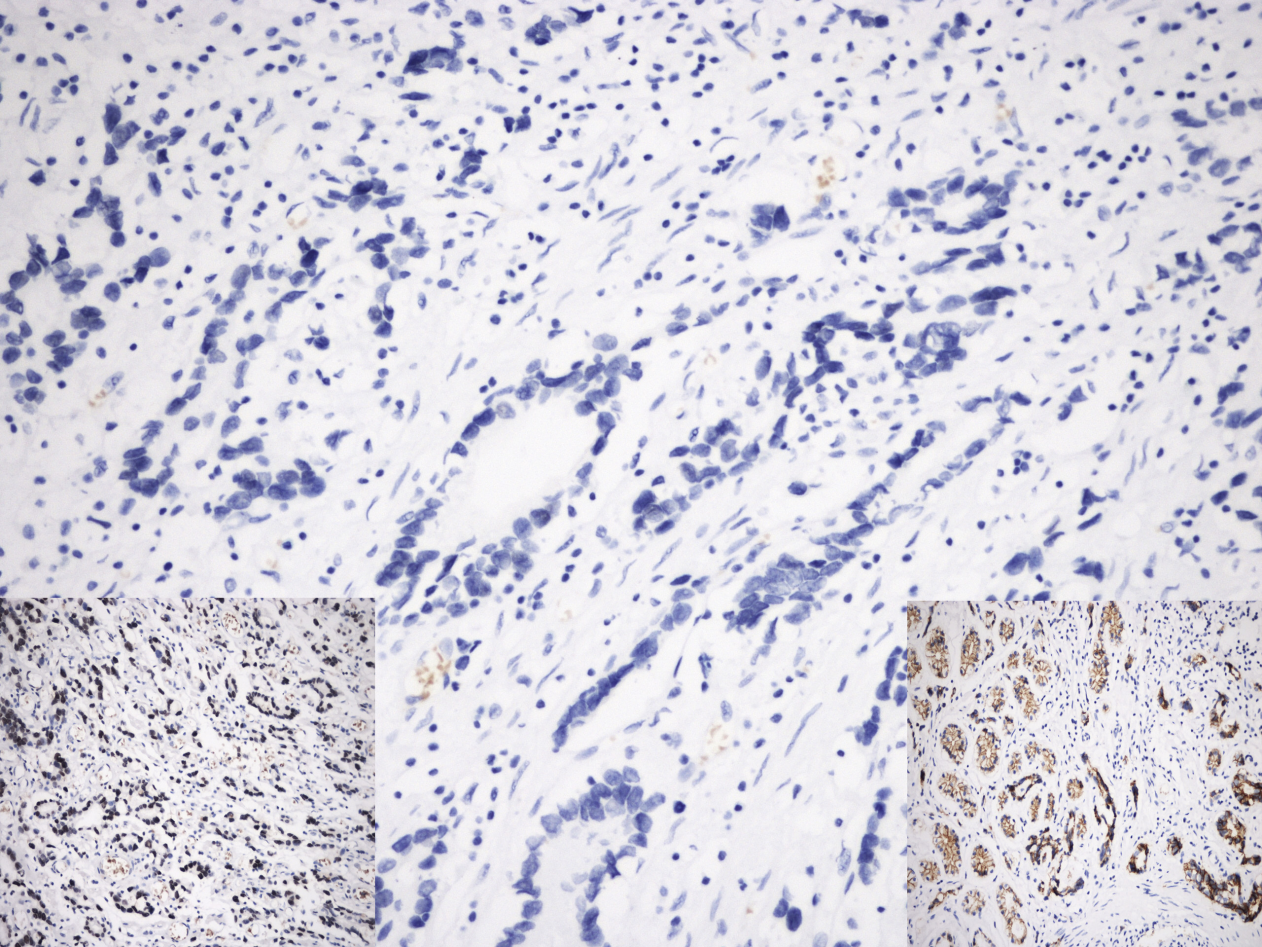
**Immunohistochemical analysis of MLH1 expression in the gastric adenocarcinoma from individual G150**. The tumor shows loss of MLH1 expression. The MSH2 and E-cadherin proteins were normally expressed in the same tumor (insets to the left and right lower fields, respectively).

The *MLH1 *2101C>A mutation was identified in eight patients. Carcinoma of one mutation carrier (individual G150) was available for IHC testing, and showed loss of MLH1 protein expression with an absence of detectable nuclear staining. The MSH2 protein was normally expressed in the same tumor with normal positive nuclear staining. IHC of E-cadherin also showed positive staining (normal membrane staining of tumor cells) (Figure 1).

## Discussion

It is widely accepted that genetic and environmental factors may be important in the etiology of gastric cancer. Among the genetic factors, *MLH1*, an important gene for DNA mismatch repair that is associated with various cancers, could play a role in the development of gastric cancer. Moreover, there might be an ethnic difference in terms of *MLH1 *mutation frequency between Eastern Asians and Western populations.

In the current study, the coding and promoter core regions of *MLH1 *were studied to achieve a full spectrum of *MLH1 *germline mutations in Chinese gastric cancer patients. Six different mutations were identified including two promoter region alterations, -93 G>A and -28A>G, and four missense mutations, 649C>T (R217C), 655 A>G (I219V), 1151T>A (V384D) and 2101C>A (Q701K).

The *MLH1 *-93 G>A single nucleotide polymorphism (SNP) is located in the core promoter region, the 93rd nucleotide upstream of the translation start site, and has been described in a broad range of ethnic backgrounds. This study revealed a frequency of 59% in control subjects for the -93 A allele, consistent with the frequency reported in Eastern Asians [7-9], but much higher than the 20% prevalence reported for Europeans [10-13]. This suggests a possible ethnic difference in terms of the frequency of this polymorphism. The -93 G>A SNP in *MLH1 *has been implicated in the etiology of various human cancers including breast, endometrial and ovarian cancer [8,10,12]. It is reported to have no effect on susceptibility to sporadic colorectal cancers (CRC) [13], but when it is classified according to MSI status, this variant is significantly associated with an increased risk of microsatellite-unstable CRC [11]. Furthermore, the -93 G>A polymorphism has been shown to be associated with *MLH1 *methylation, CpG island methylator positive phenotype, and BRAF V600E mutation in microsatellite unstable CRC [14]. It is significantly associated with the risk of lung squamous cell carcinoma with a gene-smoking interaction [7] but not with the risk of early-onset lung cancer [9]. In the present study, the frequency of the -93 A allele was not significantly different between gastric cancer cases and controls, suggesting that this *MLH1 *SNP is not associated with gastric cancer in our Chinese population.

The *MLH1 *-28A>G variation was first detected in a Chinese female with endometrial and ovarian cancer [15], and was reported as being present in 1/85 Chinese colorectal cancer patients with a family history of cancer [16]. Furthermore, its presence has been demonstrated in Finnish prostate cancer and Portuguese HNPCC patients [17,18]. The current study provides the first exact frequency of the *MLH1 *-28A>G variation in Chinese gastric cancer cases, namely 1.7% (4/236), and in controls (4/240 (1.7%); *P *= 1.000). All the variation carriers were heterozygous for this alteration. It is possible that one polymorphism can increase the effect of other related polymorphisms of a gene. Therefore, the two SNPs in the promoter region were combined to form a series. However, no association was demonstrated between cases and controls for the different combination of the two SNPs (Table 4).

*MLH1 *649C>T (R217C) was first detected in a Japanese patient suffering from hereditary nonpolyposis colorectal cancer (HNPCC) [19]. In the current study, *in silico *analysis predicted *MLH1 *649C>T to damage protein function. However, although a higher frequency of *MLH1 *649 CT genotype was detected in gastric cancer patients than controls (5/236 and 1/240, respectively), the differences did not achieve significance (*P *= 0.120, Table 2), which raises a question about its pathogenicity. However, we do realize the limitation of this study due to the relatively small sample size.

*MLH1 *655 A>G (I219V) has been reported as a common polymorphism in Western populations, with a G allele frequency of more than 30% [11,20,21]. However, in the current study it was only detected in 7 of 236 gastric cancer patients and in 12 of the 240 healthy individuals. In gastric cancer patients the G allele frequency was 1.5%, lower than in controls who demonstrated a frequency of 2.5%, similar to data in Eastern Asians where the G allele frequency is reported to be approximately 2% [8,21]. This is indicative of an ethnic difference in the frequency of this polymorphism.

*MLH1 *1151T>A V384D has been frequently detected in Eastern Asian HNPCC patients [15,16,22], but its etiology in terms of cancer has not been elucidated. This study demonstrates a comparable frequency of the variant genotype in gastric cancer cases and controls, suggesting that it might not be associated with gastric cancer.

*MLH1 *2101 C>A (Q701K) was first identified by our group in two Chinese gastric cancer cases [23,24] and subsequently by Yap et al., in 2/85 Chinese HNPCC patients [16]. Until now, this variant has not been reported in other ethnic groups. In the current study, *MLH1 *2101 C>A was detected in 8 of 236 gastric cancer cases and in 3 of the 240 control subjects. This variant could not be associated with gastric cancer in the present study when all individuals were analyzed together (*P *= 0.120, Table 2). However, when females and males were considered separately, 2101 CA carrier males had a higher risk of gastric cancer than wild type individuals (OR = 8.42, 95% CI = 1.04-68.06, *P *= 0.041; Table 3). *MLH1 *2101 CA did not significantly influence the risk of gastric cancer in females. This result may be a reflection on the small number of women enrolled in this study, or it may indicate gender differences in terms of risk. A higher frequency of *MLH1 *2101 CA was detected in gastric cancer patients aged between 50 and 59 years (five out of 65; 7.7%) than in controls (0.0%). However, it was not significantly associated with the risk of gastric cancer (*P *= 0.069, Table 3), and only a trend was observed. All patients carrying this variation were suffering from poorly or moderately differentiated adenocarcinoma.

Our findings suggest an association between the *MLH1 *variation 2101 C>A and gastric cancer risk in males, and this may be of biological significance. *In silico *analysis and *in vitro *IP in this and a previous study [25] demonstrated that this variant would not significantly affect protein function. However, recent studies have demonstrated that missense or silent mutations may interfere with normal splicing [26-29]. As the variation is located in the last amino acid in exon 18 of the *MLH1 *gene, *in silico *analysis on splicing of pre-mRNA was performed. ESEfinder predicted that this change might influence the splicing effect of the exon in *MLH1 *and therefore affect RNA transcription and protein expression of *MLH1*. IHC analysis in index patient G150 demonstrated a loss of MLH1 protein and normal expression of MSH2 and E-cadherin (Figure 1). It could be tentatively suggested that the *MLH1 *2101 C>A variation might increase the risk of gastric cancer in males. However, we cannot exclude the possibility of an estimator bias related to the relatively small sample size and the low frequencies of variations found. Study of a larger population and functional analysis are needed for further evaluation of the role of this variant.

## Conclusions

We have provided a full variation spectrum of the *MLH1 *gene in Chinese gastric cancer patients. This data indicates that there is an ethnic difference in terms of *MLH1 *mutation frequency between Eastern Asians and Western populations. Moreover, in this case-control study concerning gastric cancer, it was found that the *MLH1 *2101 C>A mutation may contribute to an increased risk of gastric cancer in males.

## Abbreviations

*CIs*: confidence intervals; *CRC*: colorectal cancers; *HNPCC*: hereditary nonpolyposis colorectal cancer; *HRM*: high: resolution melting; *IHC: *immunohistochemistry; *OR*: odds ratio; *SIFT*: Sorting Intolerant from Tolerant; *SNP*: single nucleotide polymorphism.

## Competing interests

The authors declare that they have no competing interests.

## Authors' contributions

YMF conceived and designed the study, and drafted the manuscript. WXZ and BSX carried out mutation screening, case-control and bioinformatic analysis. LFW, NX and QH contributed to the collection of samples and clinical data. YPW reviewed and modified the paper. All authors read and approved the final manuscript.

## Pre-publication history

The pre-publication history for this paper can be accessed here:

http://www.biomedcentral.com/1471-230X/11/133/prepub

## Supplementary Material

Additional file 1 **Table S1: Primer sequences for amplification of the *MLH1 *gene**. This table contains the PCR primers used for *MLH1 *gene. Click here for file
