# Supplementary material for: The MLH1 2101C>A (Q701K) variant increases the risk of gastric cancer in Chinese males
Source: BMC Gastroenterol. 2011 Dec 3;11:133. doi: 10.1186/1471-230X-11-133 (PMC3275522; doi:10.1186/1471-230X-11-133)
Supplement: Additional file 1 — Table S1: Primer sequences for amplification of the MLH1 gene. This table contains the PCR primers used for MLH1 gene. [file 1471-230X-11-133-S1.DOC]

Additional file 1, Table S1: Primer sequences for amplification of the *MLH1* gene.

| Location | Forward Primer | Reverse Primer |
| --- | --- | --- |
| Promoter | 5'- gaagagcggacagcgatctctaac -3’ | 5'-cattttggcagaagagccaag-3’ |
| Exon 1 | 5'-aggcactgaggtgattggc-3’ | 5'-tcgtagcccttaagtgagc-3’ |
| Exon 2 | 5'-aatatgtacattagagtagttg-3’ | 5'-cagagaaaggtcctgactc-3’ |
| Exon 3 | 5'-agagatttggaaaatgagtaac-3’ | 5'-acaatgtcatcacaggagg-3’ |
| Exon 4 | 5'-aacctttccctttggtgagg-3’ | 5'-gattactctgagacctaggc-3’ |
| Exon 5 | 5'-gattttctcttttccccttggg-3’ | 5'-caaacaaagcttcaacaatttac-3’ |
| Exon 6 | 5'-gggttttattttcaagtacttc-3’ | 5'-gctcagcaactgttcaatgtatg-3’ |
| Exon 7 | 5'-ctagtgtgtgtttttggc-3’ | 5'-cataaccttatctccacc-3’ |
| Exon 8 | 5'-ctcagccatgagacaataaatcc-3’ | 5'-ggttccaaaataatgtgatgg-3’ |
| Exon 9 | 5'-caaaagcttcagaatctc-3’ | 5'-gtgggtgtttcctgtgagtg-3’ |
| Exon 10 | 5'-catgactttgtgtgaatgtac-3’ | 5'-gaggagagcctgatagaacat-3’ |
| Exon 11 | 5'-gggctttttctccccctccc-3’ | 5'-aaatctgggctctcacg-3’ |
| Exon 12 | 5'-cctcgtcttctacttctgg-3’ | 5'-ctgggagttcaagcatctcctcatct-3’ |
| Exon 13 | 5'-tgcaacccacaaaatttggc-3’ | 5'-ctttctccatttccaaaacc-3’ |
| Exon 14 | 5'-tggtgtctctagttctgg-3’ | 5'-cattgttgtagtagctctgc-3’ |
| Exon 15 | 5'-cccattttgtcccaactgg-3’ | 5'-cgatcagttgaaatttcag-3’ |
| Exon 16 | 5'-catttggatgctccgttaaag-3’ | 5'-cacccggctggaaattttatttg-3’ |
| Exon 17 | 5'-ggaaagcactggagaaat-3’ | 5'-ccctccagcacacatgcatgta-3’ |
| Exon 18 | 5'- taagtagtctgtgatctccg-3’ | 5'-ctagtcctggggtgccagtgtg-3’ |
| Exon 19 | 5'-gacaccagtgtatgttgg-3’ | 5'-agagaaagaagaacacatccc-3’ |
